# Predicting Adolescents’ Problematic Social Media Use From Profiles of Internet-Specific Parenting Practices and General Parenting Dimensions

**DOI:** 10.1007/s10964-023-01816-4

**Published:** 2023-07-04

**Authors:** Suzanne M. Geurts, Ina M. Koning, Regina J. J. M. Van den Eijnden, Helen G. M. Vossen

**Affiliations:** 1 Department of Interdisciplinary Social Science, Utrecht University, Utrecht, The Netherlands; 2 Department of Educational and Family Studies, VU Universiteit Amsterdam, Amsterdam, The Netherlands; 3 Department of Clinical Child and Family Studies, Utrecht University, Utrecht, The Netherlands

**Keywords:** Problematic social media use, Adolescents, General parenting, Internet-specific parenting, Latent profile analysis

## Abstract

Although both Internet-specific and general parenting have been linked to adolescents’ problematic social media use, until now they have been investigated as separate predictors of this behavior. As specific parenting practices occur in the broader general parenting context, this study examined how different Internet-specific parenting practices (Internet-specific rule setting, reactive restrictions towards Internet use, and co-use) and general parenting dimensions (responsiveness and autonomy-granting) co-occur, and act together in predicting adolescents’ problematic social media use. Four-wave data of 400 adolescents (T1: *M* age = 13.51 years, *SD* = 2.15, 54% girls) were used. Latent profile analysis identified three parenting profiles: *Limiting and less supportive* (13.5%), *Tolerant and supportive* (25.5%), and *Limiting and supportive* (60.8%). Membership to *Tolerant and supportive* predicted lower scores on prospective problematic social media use than membership to the other profiles. Besides, membership to *Limiting and supportive* predicted lower scores on problematic social media use than membership to *Limiting and less supportive*. No robust moderation effects of adolescents’ age and gender were found. These findings suggest that a supportive general parenting context rather than Internet use restrictions should be the focus when considering the prevention of adolescents’ problematic social media use.

## Introduction

As (almost) constant use of social media throughout the day has become more and more normative among adolescents (Boer et al., 2022), the issue of problematic social media use is gaining growing attention. Problematic social media use is characterized by addictive-like symptoms such as being preoccupied with social media, and showing withdrawal symptoms when trying to stop using them (Griffiths et al., 2014). Similar to other behavioral addictions, problematic social media use may harm adolescents’ physical, psychological and/or social wellbeing (e.g., Boer et al., 2021; Shensa et al., 2017; Wong et al., 2020). Therefore, parents are highly interested in how to prevent or reduce problematic use of social media among their offspring. Internet-specific parenting practices are expected to play a role in minimizing disadvantages and risks of Internet use for youth. However, studies on these practices and problematic Internet use revealed only small effects and often inconsistent findings (e.g., Bleakley et al., 2016; Van den Eijnden et al., 2010; Wu et al., 2016). These studies generally used a variable centered approach by, mainly, examining different Internet-specific parenting practices as separate predictors of problematic Internet use ignoring four important aspects: parents are likely to apply more than one Internet-specific parenting practice, the combination of practices used by parents can differ across families (Cox et al., 2021), these unique combinations of Internet-specific parenting practices may relate to problematic Internet use differently, and Internet-specific parenting practices happen within and interact with the broader general parenting style (the integrative model of Darling and Steinberg (1993) and the self-determination theory of Ryan & Deci (2000). An examination of various Internet-specific parenting practices and general parenting dimensions *together* by using a person-centered and longitudinal approach is paramount to see how these behaviors, in combination, affect adolescents’ problematic social media use. In this study, parenting profiles will be identified based on three Internet-specific parenting practices (Internet-specific rules, reactive restrictions and co-use) and the three key dimensions of parenting that define the general parenting style (responsiveness, demandingness and autonomy-granting) using latent profile analysis. Subsequently, it will be tested whether and how these profiles relate to (changes in) adolescents’ level of problematic social media use. In addition, the moderating effects of adolescents’ gender and age will be tested.

### Parenting Profiles Based on Various Internet-Specific Parenting Practices and General Parenting Dimensions

So far, knowledge on how various Internet-specific parenting practices and general parenting dimensions co-occur is limited. Previous studies that used a latent profile analysis approach either only included general parenting dimensions or only Internet-specific parenting practices. Studies conducting a latent profile analysis on general parenting dimensions exist to a wide extent and are generally based on the three key dimensions of parenting: responsiveness, demandingness and autonomy-granting. Responsiveness is the extent to which parents show affective warmth, acceptance, and emotional support. Demandingness is about placing limits and setting boundaries to guide children’s behavior and implementing consequences for disobedience (Baumrind, 1967). Autonomy-granting refers to the extent to which parents support their children’s independence by e.g., allowing and encouraging their children to make their own decisions, express their ideas and have their own opinion (Steinberg et al., 1992). These studies have resulted in the well-established four parenting styles that describe the rearing approach across different domains and situations: authoritative (high on responsiveness, demandingness and autonomy-granting), authoritarian (high demandingness, and low responsiveness and autonomy-granting), permissive (high responsiveness and autonomy-granting, and low demandingness), and negligent (low on all three dimensions; Baumrind, 1991). Studies identifying profiles based on Internet-specific parenting practices are scarce, but e.g., one study did based on device access, parental monitoring, and communication regarding online behavior (Cox et al., 2021). This study revealed five different profiles: one profile reflecting high scores on all Internet-specific parenting practices, one -profile reflected low overall scores, and the other three profiles each consisted of a unique mixture of high, low and/or moderate scores on these three Internet-specific parenting practices.

Regarding the co-occurrence of Internet-specific parenting practices and general parenting dimensions, one would expect that parents’ specific parenting might reflect their general parenting (Blissett, 2011), because the broader parenting context characterizes how parents interact with their children in general (that is, across a range of different behavioral domains; Power, 2013). For example, parents whose general parenting is characterized by high responsiveness and demandingness, might also be more supportive and controlling in the domain of Internet use. This idea is supported by some studies on the co-occurrence of general and specific parenting behaviors related to other adolescent behavior. These studies found, for example, that positive general parenting behaviors, such as high responsiveness and autonomy-granting, commonly co-occurred with favorable specific parenting practices such as modelling and encouraging healthy eating and physical activity (Berge et al., 2010; Jennings et al., 2019), while negative general parenting, that is characterized by low responsiveness, low autonomy-granting and high demandingness, commonly co-occurred with unfavorable specific parenting practices, for instance forcing the child to eat (Jennings et al., 2019). However, as a previous study showed weak correlations between Internet-specific parenting practices and general parenting style (Geurts et al., 2021), this may not necessarily be the case. Since Internet-specific parenting practices are specific to the domain of Internet use, other factors such as parents’ attitude towards Internet use and their perception of their children’s ability to control their Internet use may also influence which practices parents actually use and to what extent (Sciacca et al., 2022). Therefore, it is expected to find multiple unique combinations of Internet-specific parenting practices and general parenting dimensions, in which the first and the latter do not necessarily align with each other.

### Combined Influence of Internet-Specific Parenting Practices and General Parenting Dimensions on Problematic Social Media Use

To date no studies have conducted a latent profile analysis based on various Internet-specific parenting practices and general parenting dimensions and examined associations of these profiles with adolescents’ problematic social media use. Therefore, it is unknown which parenting profile(s) would be most effective in preventing problematic social media use, and which profile(s) place(s) adolescents more at risk for problematic social media use. Yet, a few studies demonstrated that associations between Internet-specific parenting practices and problematic Internet use depend on general parenting. For example, the negative association between restrictive mediation and problematic Internet use has been found to be stronger for adolescents reporting higher attachment, communication, and comfort at home (Chng et al., 2015). Besides, based on e.g., the integrative model of Darling and Steinberg (1993) and the self-determination theory (Ryan & Deci, 2000), it can be argued that general parenting may enhance or decrease the effectiveness of specific parenting practices through its’ effect on the extent to which a child internalizes these parenting behaviors. Such findings and theories point out the importance of taking both general as well as Internet-specific parenting into consideration. Studies addressing other adolescent behaviors have shown that different parenting profiles based on various general and/or specific parenting behaviors are uniquely related to e.g., eating behavior (Jennings et al., 2019) and substance use (Abar et al., 2014; Koning et al., 2012). For example, parents with an authoritarian or a permissive general parenting style and controlling feeding practices are more likely to have children who ate to obtain pleasure and lacked internal cues for hunger (risk factors for overweight) than parents with an authoritative parenting style and less controlling feeding practices (Jennings et al., 2019). Based on the above, it is expected that the identified parenting profiles in this study will be differentially related to (changes in) adolescents’ level of problematic social media use.

### The Moderating Role of Adolescents’ Gender and Age

It is also important to examine whether adolescents’ gender and age moderate the relationship between parenting profile and adolescents’ problematic social media use. One study showed that Internet-specific rules had a protective effect on problematic social media use, but only for girls (Koning et al., 2018). Another study found different general and Internet-specific parenting factors predicting smartphone addiction among boys than among girls (Lee & Kim., 2018). Also, a recent meta-analysis on the relationship between general and Internet-specific parenting and problematic Internet use revealed different findings for different populations. For example, a stronger positive relationship was found between restrictive mediation and problematic Internet use for adolescents older than 14 years compared to adolescents younger than 14 years (Lukayská et al., 2022). Thus, gender and age differences are expected in the effects of parenting profiles on adolescents’ problematic social media use.

## Current Study

A gap in the literature exists regarding how Internet-specific parenting practices and general parenting dimensions co-occur and act together in predicting adolescents’ problematic social media use (over time). In an attempt to capture the complexity of parenting approaches in relation to adolescents’ Internet use and its effects on adolescents’ problematic social media use, this study examines three research questions. The first research question that will be examined - using latent profile analysis - is which different parenting profiles can be distinguished based on three perceived Internet-specific parenting practices (Internet-specific rules, reactive restrictions towards Internet use and co-use) and three general parenting dimensions (responsiveness, demandingness, and autonomy-granting). The second research question is to what extent these parenting profiles predict (changes in) adolescents’ level of problematic social media use. The third research question is whether the relationships between parenting profiles and adolescents’ problematic social media use are moderated by adolescents’ gender and age. Due to the lack of knowledge on how Internet-specific rules, reactive restrictions towards Internet use, co-use, responsiveness, demandingness and autonomy-granting co-occur, and the exploratory nature of latent profile analysis, no hypotheses are formulated about the profiles that will be identified. And, therefore, no hypotheses are formulated regarding the second and third research question.

## Method

### Procedure

Adolescent data from wave 1 to 4 of the Digital Family project were used (Geurts et al., 2022). This Dutch research project investigates youth digital media use in the context of the family. Participants were recruited through multiple sources (schools, sport clubs, social media channels, word-of-mouth, and paper flyer distribution) and asked to fill in an online survey at home. Data were collected from April to July 2020 (wave 1), in November and December 2020 (wave 2), from May to July 2021 (wave 3) and from November 2021 to January 2022 (wave 4). Participants provided active informed consent at the beginning of the survey and active parental consent was given in the registration form. On average, the survey took 30 minutes to complete. A gift card of €5 was offered for each completed survey and after each wave we raffled a gift card of €100.

### Participants

A total of 400 adolescents provided data on the study variables at wave 1. During wave 2 and 3 additional participants were recruited. At wave 2, a total of 386 adolescents provided data on the study variables (of which 268 participated at wave 1), at wave 3 257 (of which 182 participated at wave 1) and at wave 4 238 (of which 176 participated at wave 1). Altogether, 564 participants took part in at least one measurement wave. Of the study sample at T1, 46% was boy, ages ranged from 9 to 19 years (*M* = 13.51, *SD* = 2.15) and almost all (96.5%) were born in the Netherlands. Regarding educational level, 25.3% was in primary school, 16% in lower, 20.8% in middle and 32.3% in higher level secondary education, 4.5% in secondary vocational education and 1.3% in higher professional education. Most participants (84%) were living with both biological parents.

Attrition analyses revealed that non-responding adolescents at T2 were more likely to be girls (χ^2^ = 4.804, *p* = 0.028). No differences were found between adolescents who completed the follow-up assessment at T2, T3 or T4 and adolescents who did not with respect to country of birth, living in a (non-)intact family and problematic social media use at T1.

### Measures

#### Adolescents’ problematic social media use

The Social Media Disorder scale (Van den Eijnden et al., 2016) was used to measure adolescents’ problematic social media use at all four waves. This scale consists of nine items based on the DSM-5 diagnostic criteria for Internet Gaming Disorder measuring the symptoms of addiction. Participants were asked whether they, in the past year: 1) often felt bad when they could not use social media (withdrawal), 2) regularly felt dissatisfied because they wanted to spend more time on social media (tolerance), 3) regularly could not think of anything else but social media (preoccupation), 4) failed to spend less time on social media (persistence), 5) regularly neglected other activities because of social media (displacement), 6) regularly had arguments with others because of their social media use (problem), 7) often used social media secretly (deception), 8) often used social media to not have to think about unpleasant things (escape), and 9) had serious conflicts with parents or siblings because of their social media use (conflict). Participant could answer these items on a dichotomous sale (0 = *no*, 1 = *yes*). The items ‘deception’ and ‘escape’ slightly deviated from the original 9-item SMD scale. Higher sum scores indicated more problematic social media use symptoms. Because of the dichotomous response scale, internal consistency was calculated using the ordinal alpha that is based on the tetrachoric correlation matrix (Gadermann et al., 2019). Tetrachoric ordinal alpha was 0.86 at T1, 0.84 at T2, 0.90 at T3 and 0.84 at T4.

#### Internet-specific rule setting

Internet-specific rule setting was assessed by asking adolescents at T1 to what extent they were allowed to (1) “use the Internet or play games as long as they wanted”, (2) “use the Internet or play games for more than three hours”, (3) “use the Internet or play games while their homework was not finished yet”, (4) “use the Internet or play games in the hour before going to sleep”, (5) “bring their smartphone or Tablet to their bedroom when going to sleep at night”, (6) “keep their smartphone or Tablet with them while doing homework”, (7) “keep their smartphone or Tablet with them during dinner” and (8) “keep on using their smartphone or Tablet while talking with their parents” in the past two weeks (Geurts et al., 2022). They answered these questions on a scale from 1 (*never*) to 5 (*very often*). Items were reversed coded so that higher mean scores indicated stricter Internet-specific rule setting. Cronbach’s alpha was 0.80.

#### Reactive restrictions towards Internet use

Reactive restrictions was measured by asking adolescents at T1 to think about the past two weeks and answer the following questions: “When you want to (keep on) using the Internet or play games, how often do your parents react that…” (1) “…you are not allowed to use the Internet or play games?”, (2) “…you are only allowed to use the Internet or play a game for a short period of time?”, (3) “…you have a certain time (e.g., 5 minutes) to use the Internet or play a game?” (4) “…you have to turn off the computer/Tablet or smartphone?” (Koning et al., 2018). The items included a 5-point response scale ranging from 1 (*(hardly) ever*) to 5 (*more than 5 times a day*). Higher mean scores indicated more reactive restrictions towards Internet use. Cronbach’s alpha was 0.84.

#### Co-use

For co-use, adolescents indicated at T1 how often they spend time with their parents doing the following activities: (1) watching television, a movie, series or vlogs together, (2) playing an online game together, (3) making a vlog together?”. Response options included 1 (*not once*), 2 (*less than once a week*), 3 (*once a week*), 4 (*a few times a week*), 5 (*several times a week*) and 6 (*(almost) every day*). Higher sum scores indicated more co-use. Internal consistency was not assessed since it entails a formative scale (Diamantopoulos & Winklhofer, 2001).

#### Responsiveness

Responsiveness was measured by using three items of the Parenting Style Inventory II (Darling & Toyokawa, 1997) which were answered on a 5-point scale ranging from 1 (*totally disagree*) to 5 (*totally agree*). The three items were: (1) “I can count on my parents or caregivers to help me out if I have a problem”, (2) “My parents or caregivers hardly ever praise me for doing well”, (3) “My parents or caregivers and I do things that are fun together”. Item two was reversed coded so that higher mean scores indicated higher levels of responsiveness. Cronbach’s alpha at T1 was 0.63.

#### Demandingness

Demandingness was assessed at T1 with the following four items of the Parenting Style Inventory II (Darling & Toyokawa, 1997): (1) “My parents or caregivers expect me to follow family rules”, (2) “My parents or caregivers let me get away with things”, (3) “If I do not behave myself, my parents or caregivers will punish me” and (4) “My parents or caregivers point out ways I could do better”. However, Cronbach’s alpha was unacceptably low (0.33) and did not improve when removing an item. This variable was excluded.

#### Autonomy-granting

Autonomy-granting was assessed at T1 with the following items on a 5-point scale ranging from (*totally disagree*) to 5 (*totally agree*): (1) “My parents or caregivers respect my privacy”, (2) “My parents or caregivers give me a lot of freedom”, (3) “My parents or caregivers make most of the decisions about what I can do”, (4) “My parents or caregivers believe I have a right to my own point of view” (Darling & Toyokawa, 1997). Item three was reversed coded so that higher mean scores indicated higher levels of autonomy-granting. Cronbach’s alpha was 0.69.

#### Age

Adolescents’ age in years was calculated using their date of birth and date of participation at T1.

#### Gender

Adolescents indicated being a boy (0) or a girl (1).

### Analysis Plan

Multiple latent profile analyses were performed to identify profiles based on perceived general and Internet-specific parenting behaviors at T1 (RQ 1). Latent profile analysis is a person-centered approach that classifies individuals into distinct profiles, each profile consisting of individuals who show a similar response pattern on a certain set of continuous indicators. As indicators, responsiveness, autonomy-granting, Internet-specific rule setting, reactive restrictions towards Internet use and co-use measured at wave 1 were included. Note that demandingness was not included, since the reliability of this measure was too low. These variables were standardized to account for differences in scales. To determine the optimal number of profiles, a series of latent profile analysis with increasing number of profiles were fitted, starting with a single-profile model. Adequacy of fit of these models was compared using multiple criteria. The first one is the Bayesian Information Criterion (BIC). The lower the value of BIC, the better the model fit. In addition, Lo-Mendell-Rubin adjusted likelihood ration test (LMR-LRT) and bootstrap likelihood ration test (BLRT) were evaluated. *p*-values < 0.05 indicate improved model fit of the current model compared to the model with one profile less (Masyn, 2012). Furthermore, entropy values were evaluated as an indication of assignment accuracy. Values closer to 1 indicate more accurate classification (Van de Schoot et al., 2017). Besides, profile sizes will be taken into account (a minimum of 5% of the total sample size). Lastly, distinctiveness and theoretical meaningfulness of the different profiles will be considered (Bauer, 2022). For subsequent analyses, the most likely class membership variable was used to create dummy variables to represent the latent profiles.

To examine whether the identified profiles predicted (change in) adolescents’ problematic social media use (RQ 2), an unconditional (without predictors) latent growth curve model (LGCM) was used to examine the change in problematic social media use. Four measurement waves of problematic social media use with approximately equal time intervals over a 1.5-year period were included. Model fit was compared of an intercept-only model (no growth), a linear growth model and a quadratic growth model. As the unconditional LGCM showed no significant change over time in problematic social media use (see model fit indices in Results section), instead of running a conditional LGCM subsequently, multiple regression models were conducted to examine associations between the identified parenting profiles and problematic social media use at T1, T2, T3 and T4. In the analyses were controlled for age (*in years*), gender (0 = *boy*, 1 = *girl*), family intactness (0 = *intact*, 1 = *non-intact*) and parental educational level (mean score of both parents’ (if available) highest educational attainment) as these demographic characteristics have been linked to parenting and/or problematic social media use (e.g., Andreassen et al., 2017; Özgür, 2016; Su et al., 2020). To compare all latent profiles, three dummy variables were created. In each regression model, a different dummy variable was used as predictor.

To examine whether adolescents’ gender and age moderated the associations between parenting profiles and adolescents’ problematic social media use, interaction variables between the dummy variables and gender/age were included in the models. To compute the interaction variables with age, age was centered. Each interaction effect was tested in a separate model. Because of the large number of tests (24 interaction effects), Bonferroni correction was applied for the moderation analyses. Accordingly, for these analyses a *p*-value of < 0.002 was regarded as significant.

As problematic social media use is a count variable with a positively skewed distribution, the multiple regressions for RQ 2 are actually Poisson models. Maximum likelihood estimation with robust standard errors (Múthen & Múthen, 2017) was applied to account for the clustered nature (siblings) of the data and full information maximum likelihood was applied to deal with missing data. In the latent profile analyses and the analyses predicting problematic social media use (main- and interaction-effects), participants who did not participate at wave 1 were excluded.

Finally, the influence of careless/inattentive responders on the results was investigated by rerunning the analyses using the sample excluding participants being flagged as careless/inattentive responder. Careless responders were identified per wave by looking at response invariability (using scales for which non-varying answers are not plausible), response inconsistency (using psychometrically synonymous item pairs (a cut-off score of *r* > 0.50 was used)) and an instructed response item to check attention (this item was included somewhere halfway the survey in wave 3 and wave 4). Participants were flagged as careless responder when at least one of these methods gave an indication of random or inattentive responding. The analyses were preregistered in Open Science Framework (website omitted for double anonymized review purpose).

## Results

### Latent Profile Analysis

#### Profile solution

Multiple latent profile models were performed up to a five-profile solution. Model fit indices and classification accuracy of these models are displayed in Table 1. Since the BLRT *p*-value was significant in all profile solutions and the LMR-LRT *p*-value in none of the solutions, it was not possible to rely on these model fit indices to decide on the number of profiles. The BIC values decreased up to the five-latent-profile model. However, the five-profile solution included a profile that contained less than 5% of the total sample size. The four-profile solution included a profile that contained 6% of the total sample size. Given the total sample size (*N* = 400), this profile size was also considered as being too small. Therefore, the three-profile solution was preferred and considered the best fit to the data. This profile solution showed distinct and theoretically meaningful profiles and good assignment accuracy. On average, those who were assigned to profile 1, had a 89% chance of belonging in this profile. For profiles 2 and 3, these percentages were 85% and 91% respectively.

**Table 1:** Model fit indices and classification accuracy for different profile solutions

No. of profiles	Smallest profile size	BIC	LMR-LRT *p*-value	BLRT *p-*value	Entropy
1	400 (100%)	5724.317	-	-	-
2	48 (12%)	5636.683	0.099	< 0.001	0.830
3	47 (11%)	5575.204	0.079	< 0.001	0.752
4	24 (6%)	5558.548	0.195	< 0.001	0.768
5	7 (1,8%)	5537.979	0.330	< 0.001	0.815

*BIC* Bayesian information criterion, *LMR-LRT* Lo-Mendell-Rubin likelihood ratio test, *BLRT* Bootstrap likelihood ratio test

#### Profile interpretation

**Fig. 1:**
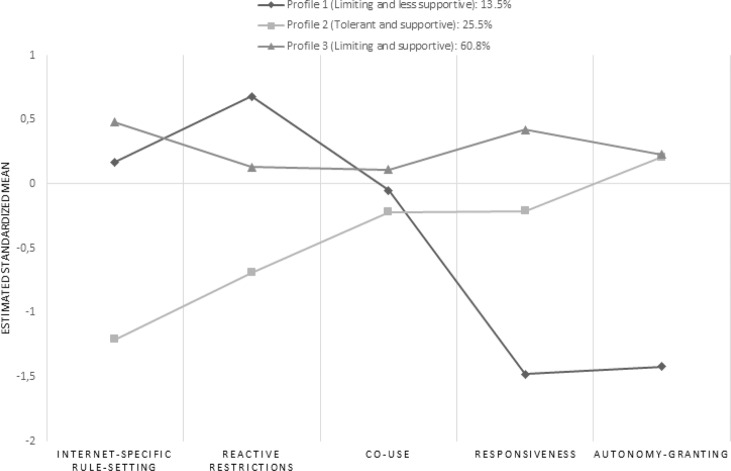
Latent profiles of Internet-specific and general parenting variables

The three parenting profiles are graphically illustrated in Fig. 1 using the estimated standardized means. Profile 1 (*Limiting and less supportive*) was the smallest (*n* = 47, 13.5%) and was characterized by highest scores on reactive restrictions, relatively high scores on Internet-specific rule setting and lowest scores on responsiveness and autonomy-granting. Profile 2 (*Tolerant and supportive*; *n* = 104, 25.5%) was characterized by lowest scores on Internet-specific rule setting and reactive restrictions, relatively high scores on responsiveness and highest scores (together with profile 3) on autonomy-granting. Profile 3 (*Limiting and supportive*) was the largest (*n* = 249, 60.8%) and was characterized by highest scores on Internet-specific rule setting, relatively high scores on reactive restrictions and highest scores on responsiveness and autonomy-granting. Co-use did not meaningfully distinguish between profiles. In Table 2, the sample means on each indicator per profile are displayed.

**Table 2:** Sample means and standard deviations on indicators across parenting profiles

Demographic characteristics	Profile 1 (Limiting and less supportive) *n* = 47	Profile 2 (Tolerant and supportive) *n* = 104	Profile 3 (Limiting and supportive) *n* = 249
Internet-specific rule setting	3.63 (0.68)	2.44 (0.51)	3.96 (0.53)
Reactive restrictions towards Internet use	2.44 (0.92)	1.29 (0.37)	1.98 (0.78)
Co-use	6.55 (2.51)	6.10 (1.84)	6.82 (1.59)
Responsiveness	3.36 (0.49)	4.19 (0.52)	4.57 (0.40)
Autonomy-granting	3.13 (0.72)	4.34 (0.51)	4.32 (0.54)

Standard deviations are in parentheses

#### Demographic characteristics across profiles

Table 3 shows the distribution of demographic characteristics across profiles. Adolescents in profile 2 (*Tolerant and supportive*) had a significantly higher mean age than adolescents in profile 1 (*Limiting and less supportive*) and profile 3 (*Limiting and supportive*). Besides, profile 3 (*Limiting and supportive*) showed a significantly higher percentage of intact families than profile 2 (*Tolerant and supportive*). Gender, parental educational level and the average number of having (older and younger) siblings did not significantly differ between profiles.

**Table 3:** Demographic characteristics across parenting profiles

Demographic characteristics	Profile 1 *n* = 47	Profile 2 *n* = 104	Profile 3 *n* = 249
Age *M* (*SD*)	13.55^a^ (2.25)	15.20 (1.43)	12.80^a^ (2.00)
Gender (% boy)	57.4%	39.5%	47.4%
Parental educational level (% highly educated)	55.3%	58.7%	61.4%
Living in intact family (% intact)	87.2%	73.1%	88%^a^
Number of siblings *M* (*SD*)	1.84 (0.95)	1.77 (1.54)	1.72 (1.23)
Number of older siblings *M* (*SD*)	0.87 (0.94)	0.95 (0.65)	0.89 (1.28)
Number of younger siblings *M* (*SD*)	0.98 (0.78)	0.82 (0.78)	0.83 (0.78)

^a^Significantly different from Profile 2

### Unconditional Latent Growth Curve Model of Adolescents’ Problematic Social Media Use

Table 4 shows results of the unconditional latent growth curve Poisson model including problematic social media use T1-T4. The intercept-only model fitted the data best, indicating no change over time in problematic social media use.

**Table 4:** Model fit of intercept only, linear and quadratic Poisson growth model

Model	AIC	BIC
Intercept-only	4047.850	4056.517
Linear model	4050.496	4072.162
Quadratic model	4054.240	4093.240

*AIC* Akaike information criterion, *BIC* Bayesian information criterion

### Associations Between Parenting Profiles and Adolescents’ Problematic Social Media Use

While controlling for adolescents’ age, gender, family intactness and parental educational level, Poisson regression models with problematic social media use at T1 as outcome variable showed that adolescents in profile 3 (*Limiting and supportive*) scored lower on problematic social media use at T1 than adolescents in profile 1 (*Limiting and less supportive*). The standardized beta coefficient indicated small effect size. The dummy variables ‘profile 3 versus profile 2’ and ‘profile 1 versus profile 2’ did not significantly relate to problematic social media use at T1 (see Table 5).

**Table 5:** Results of Poisson regression models predicting adolescents’ problematic social media use at T1, T2, T3 and T4

	Problematic social media use T1				Problematic social media use T2				Problematic social media use T3				Problematic social media use T4			
	B	SE	*β*	*p*	B	SE	*β*	*p*	B	SE	*β*	*p*	B	SE	*β*	*p*
**Model 1**																
Profile 3 ( = ref) v. profile 1	**0.641**	**0.166**	**0.234**	**0.000**	**0.506**	**0.213**	**0.185**	**0.017**	0.361	0.235	0.132	0.124	**0.641**	**0.267**	**0.234**	**0.016**
Age	−0.086	0.049	−0.161	0.078	−0.060	0.034	−0.124	0.080	−0.055	0.044	−0.114	0.212	−0.003	0.056	−0.006	0.958
Gender (boys = ref)	0.164	0.155	0.081	0.292	0.239	0.147	0.119	0.105	0.240	0.204	0.120	0.240	0.348	0.212	0.174	0.100
Family intactness (intactness = ref)	0.204	0.195	0.085	0.296	0.235	0.241	0.077	0.330	0.027	0.342	0.009	0.937	0.345	0.245	0.113	0.159
Parental educational level	**−0.238**	**0.068**	**−0.202**	**0.001**	−0.094	0.120	−0.068	0.433	−0.102	0.101	−0.073	0.313	−0.131	0.085	−0.095	0.121
**Model 2**																
Profile 1 ( = ref) v. profile 2	−0.382	0.203	−0.177	0.061	−0.380	0.235	−0.176	0.107	**−0.895**	**0.329**	**−0.414**	**0.006**	**−0.897**	**0.307**	**−0.415**	**0.004**
Age	−0.086	0.049		0.078	**−0.121**	**0.056**	**−0.228**	**0.030**	−0.085	0.075	−0.159	0.261	−0.112	0.070	−0.211	0.109
Gender (boys = ref)	0.164	0.155		0.292	0.053	0.226	0.026	0.815	0.386	0.320	0.192	0.227	0.448	0.226	0.223	0.047
Family intactness (intactness = ref)	0.204	0.195		0.296	0.297	0.281	0.124	0.291	0.099	0.338	0.041	0.771	0.013	0.357	0.005	0.972
Parental educational level	**−0.238**	**0.068**		**0.001**	0.099	0.189	0.085	0.600	−0.056	0.147	−0.048	0.704	−0.259	0.139	−0.220	0.063
**Model 3**																
Profile 3 ( = ref) v. profile 2	0.012	0.152	0.006	0.935	−0.044	0.200	−0.020	0.826	**−0.595**	**0.266**	**−0.271**	**0.025**	**−0.713**	**0.278**	**−0.325**	**0.010**
Age	0.026	0.031	0.056	0.404	−0.034	0.036	−0.073	0.337	−0.035	0.045	−0.074	0.447	0.082	0.056	0.176	0.147
Gender (boys = ref)	**0.290**	**0.126**	**0.144**	**0.021**	**0.429**	**0.146**	**0.214**	**0.003**	0.354	0.214	0.176	0.098	**0.485**	**0.222**	**0.241**	**0.029**
Family intactness (intactness = ref)	**0.317**	**0.137**	**0.118**	**0.020**	0.222	0.233	0.082	0.342	−0.042	0.279	−0.015	0.881	0.345	0.270	0.128	0.200
Parental educational level	−0.094	0.072	−0.067	0.195	−0.111	0.111	−0.080	0.320	−0.090	0.106	−0.065	0.398	−0.144	0.106	−0.104	0.173

Significant effects are in bold

**Fig. 2:**
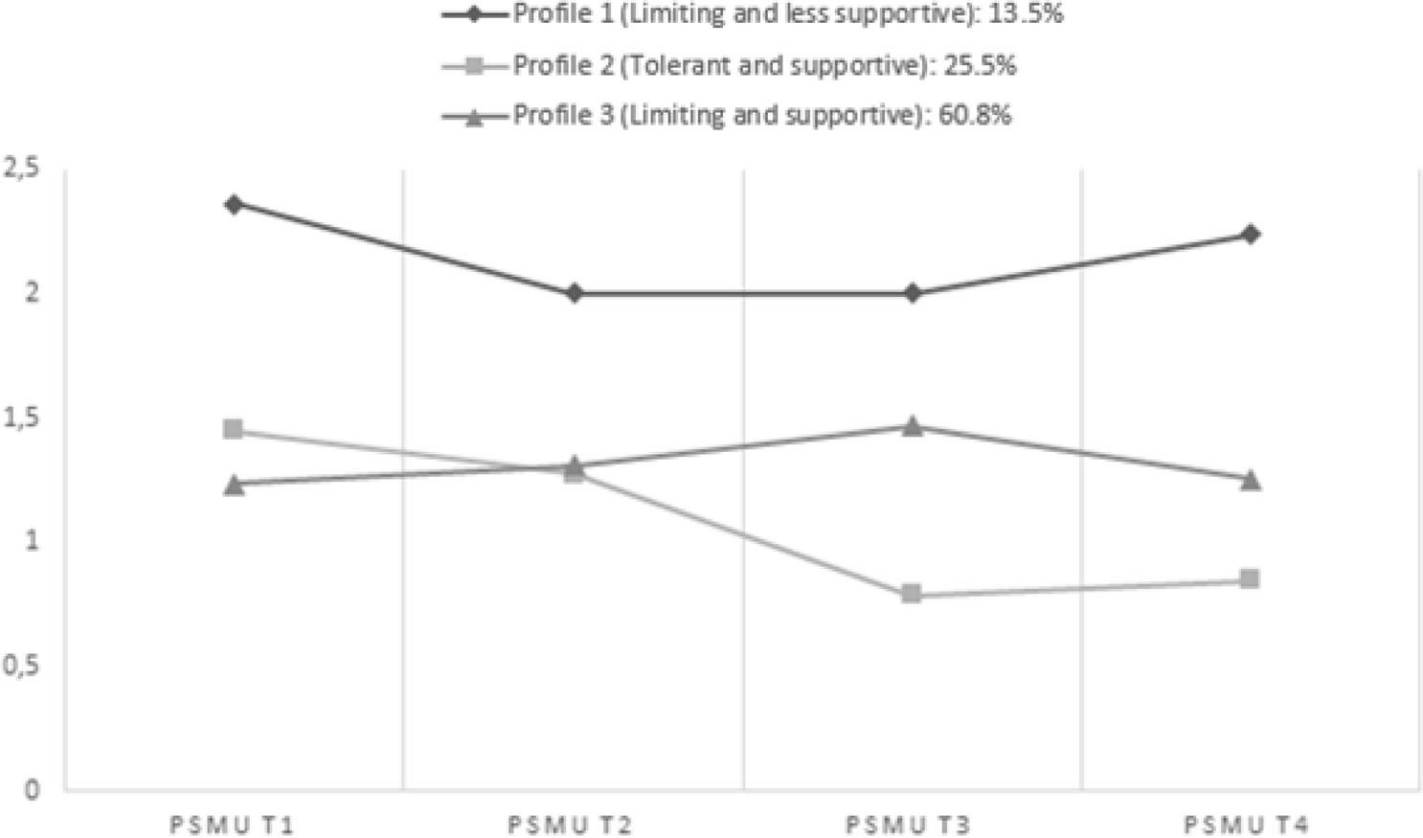
Means on adolescents’ problematic social media use across waves per profile

Longitudinal Poisson regression models with problematic social media use at a later wave as outcome variable showed significant effects of all three dummy variables (‘profile 3 versus profile 2’, ‘profile 3 versus profile 2’ and ‘profile 1 versus profile 2’) while controlling for adolescents’ age, gender, family intactness and parental educational level (see Table 5). Adolescents in profile 3 (*Limiting and supportive*) scored lower on problematic social media use at T2 and T4 than adolescents in profile 1 (*Limiting and less supportive*). Standardized beta coefficients indicated small effect sizes. Besides, adolescents in profile 2 (*Tolerant and supportive*) scored lower on problematic social media use at T3 and T4 than adolescents in profile 1 (*Limiting and less supportive*). Standardized beta coefficients indicated medium effect sizes. Furthermore, adolescents in profile 2 (*Tolerant and supportive*) scored lower on adolescents’ problematic social media use at T3 and T4 than adolescents in profile 3 (*Limiting and supportive*). Standardized beta coefficients indicated small (at T3) and medium (at T4) effect sizes. The means on problematic social media use across the four waves per profile are displayed in Fig. 2.

### Adolescents’ Gender and Age as Moderators

After applying Bonferroni correction, no significant interaction effects of adolescents’ gender and age were found.

### Sensitivity Analyses without Adolescents Identified as Careless Responders

**Fig. 3:**
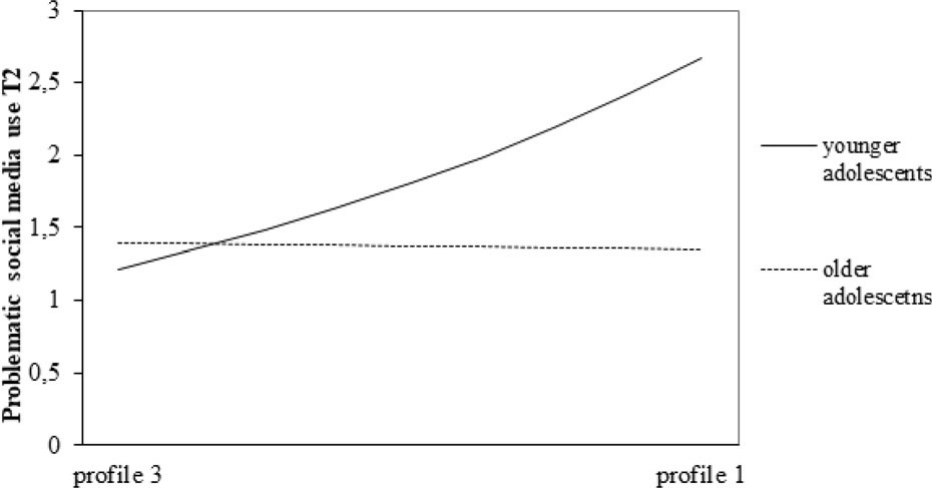
Interaction effect between ‘profile 3 v. profile 1’ and age for problematic social media use at T2

Fourteen adolescents were identified as careless responder at wave 1, seven at wave 2, eighteen at wave 3 and seventeen at wave 4. Running the analyses after removing data of these adolescents yielded two findings that deviated from the initial results. The dummy variable ‘profile 3 versus profile 1’ did no longer significantly relate to problematic social media use at T2 (B = 0.398, *SE* = 0.250, *β* = 0.143, *p* = 0.112). Besides, the interaction between the dummy variable ‘profile 3 v. profile 1’ and age was significant for problematic social media use at T2 (B = −0.278, *SE* = 0.080, *β* = −0.245, *p* < 0.001, see Fig. 3). Younger adolescents in profile 3 (*Limiting and supportive*) scored lower on problematic social media use at T2 than younger adolescents in profile 1 (*Limiting and less supportive*; B = 1.100, *SE* = 0.301, *p* < 0.000). This effect was not found for older adolescents.

## Discussion

Both Internet-specific and general parenting have been separately linked to adolescents’ problematic social media use. This study expanded the existing, mainly cross-sectional, literature on the relationship between parenting and adolescents’ problematic social media use by taking into account that parents are likely to apply multiple Internet-specific parenting practices, and that these practices happen within and interact with the broader general parenting context. Using a person-centered and longitudinal approach, adolescents’ problematic social media use was predicted from profiles of Internet-specific rule setting, reactive restrictions towards Internet use, co-use, general responsiveness and general autonomy-granting. The findings suggest that not necessarily a certain combination of Internet-specific and general parenting practices, but rather a supportive general parenting context independent from limiting adolescents’ Internet use plays a pivotal role in preventing the risk of problematic social media use.

### Profiles

The first aim was to identify profiles based on three Internet-specific parenting practices (Internet-specific rule setting, reactive restrictions towards Internet use and co-use) and three general parenting dimensions (responsiveness, demandingness and autonomy-granting). However, as the items used to measure demandingness showed very poor internal consistency, this variable was excluded. Three profiles were identified. Profile 1, the smallest group (13.5%), entitled *Limiting and less supportive*, was characterized by relatively high scores on Internet-specific rules and reactive restrictions towards Internet use, and relatively low scores on responsiveness and autonomy-granting. Profile 2 (25.5%), entitled *Tolerant and supportive*, was characterized by the lowest scores on Internet-specific rules and reactive restrictions towards Internet use, relatively high scores on responsiveness and highest scores (together with profile 3) on autonomy-granting. Finally, profile 3, the largest group (60.8%), entitled *Limiting and supportive* was characterized by highest scores on Internet-specific rule setting, relatively high scores on reactive restrictions, and highest scores on responsiveness and autonomy-granting. Within each profile there was not much variation between the different parenting behaviors within Internet-specific and general parenting. For example, when parents scored higher on Internet-specific rules, they were also likely to score higher on reactive restrictions towards Internet use, implying that when parents want to limit their child’s Internet use, they will do so in multiple ways. Therefore, the distinction between profiles was based on the level of Internet-specific versus general parenting. That is, the profiles differed in the extent to which adolescents perceived their parents as strict in limiting their Internet use via rules and reactive restrictions (co-use did not distinguish between the profiles), and in the extent to which adolescents experienced a supportive general parenting context described by responsiveness and autonomy-granting.

The profiles differed in terms of adolescents’ age and family structure. Profile 2 (*Tolerant and supportive*) consisted of significantly older adolescents than the other two profiles. This is in line with previous studies showing a negative association between adolescents’ age and restrictive mediation (Glatz et al., 2018; Navarro et al., 2022; Sonck et al., 2013; Vaala and Bleakley (2015)). This may be explained by parents shifting from restrictions to discussions and granting autonomy or parents giving up/feeling helpless due to their shown or perceived inability to regulate their child’s Internet use (Steinfeld, 2021). Profile 3 (*Limiting and supportive*) consisted of significantly more adolescents living in an intact family than adolescents in profile 2 (*Tolerant and supportive*). Perhaps it is easier to limit the Internet use of adolescents’ within intact families, as divorced parents may deal with their children’s Internet use in a different way which could make it harder to set (consistent) rules. The profiles did not differ in terms of gender, parental educational level and number of siblings.

### Profiles in Relation to Adolescents’ Problematic Social Media Use

The second aim was to examine how the identified parenting profiles predicted (changes in) adolescents’ problematic social media use. The findings showed that problematic social media use was stable; no significant change in adolescents’ problematic social media use over a time period of one and a half year could be detected. This is in line with a previous study showing that the level of problematic social media use is quit persistent over time (Boer et al., 2022). It should be noted, however, that although no significant changes were found on group level, there may have been changes over time in problematic social media use within individuals (Boer et al., 2019; Boer et al., 2021). Besides, it could be that a time span of one and a half year is too short to capture change over time.

As there was no change in adolescents’ problematic social media use to predict, it was examined whether profile membership was cross-sectionally and prospectively related to adolescents’ level of problematic social media use while controlling for adolescents’ age, gender, family intactness and parental educational level. Adolescents in the *Limiting and supportive* profile scored lower on both concurrent and prospective problematic social media use than adolescents in the *Limiting and less supportive* profile. As the level of Internet-specific rules and reactive restrictions towards Internet use were fairly the same in both profiles, this finding indicates that limiting Internet use seems to be more effective when adolescents perceive their parents as more responsive and encouraging, which is in line with the study of Chng et al. (2015), the integrative model of Darling and Steinberg (1993) and the self-determination theory (Ryan & Deci, 2000). This might be explained by the fact that adolescents are more likely to internalize/adhere to rules when receiving more parental warmth and support (Trinkner et al., 2012). Additionally, adolescents in the *Tolerant and supportive* profile were found to score lower on problematic social media use later in time than adolescents in the other profiles. Taking these findings together, the current results seem to imply that a supportive general parenting context could be more critical in order to prevent adolescents’ problematic social media use than trying to limit adolescents’ Internet use via rules and reactive restrictions. This is in line with previous research that has shown stronger relations between general parenting dimensions and problematic social media use than between Internet-specific parenting practices and problematic social media use (Geurts et al., 2022; Lukavská et al., 2022). However, this finding is in contrast with the theory that behavior-specific factors are more strongly linked to the respective behavior than general factors (Niermann et al., 2018; Power et al., 2013) for which evidence has been found in e.g., research on parenting in relation to other risk behavior such as substance use (Kokotovič et al., 2022; Van Zundert et al., 2006; Vermeulen-Smit et al., 2015). This contrast may be explained by adolescent social media use being generally accepted as normative behavior and having not only negative, but also positive effects on adolescents’ development and well-being (e.g., Uhls et al., 2017). Moreover, also different from previously studied risk behaviors, social media use takes place 24/7 without any actual age limits. Thus, as social media are an integrated, indispensable part of adolescents’ daily life parents can limit their children’s social media use to some extent but withholding them from using social media at all is not realistic/desirable. Besides, parents can have a self-interest in the digital media use of their children (Geurts et al., 2021), which is also specific for digital media use. For these reasons, preventing problematic social media use may require a different parenting approach than preventing other adolescent risk behaviors. Also, it has been stated that it is not the amount of time spend online that makes Internet use problematic (as shown by Boer et al., 2021), but the underlying reasons that drive individuals to engage in Internet use (Popadić et al., 2020). This could explain the finding that the general parenting context seems to play a bigger potential role in preventing adolescents’ problematic social media use than Internet-specific rules and reactive restrictions towards Internet use. Whereas rules and reactive restrictions are aimed at limiting the time and moments of going online (i.e. access), a supportive general parenting context may prevent adolescents from becoming dependent on social media to satisfy unsatisfied psychological needs. Following the model of compensatory Internet use (Kardefelt-Winther, 2014) and the compensatory satisfaction theory (Liu et al., 2016), when adolescents’ needs for e.g., relatedness and autonomy are not satisfied by their parents, they may become dependent on social media - that provide unrestricted freedom to i.e., interact with others – more easily to fulfill those psychological needs. Several studies have shown a link between (online) psychological needs satisfaction and problematic social media use (e.g., Kozan et al., 2019)/Internet use (e.g., Li et al., 2016; Liu et al., 2016)/smartphone use (e.g., Gao et al., 2022; Sun et al., 2020). More research on relevant mechanisms underlying the relationship between parenting behaviors and adolescents’ problematic social media use is needed. Besides, future studies should examine whether the finding implying general parenting being more crucial than Internet-specific parenting also holds for other Internet-specific parenting practices than the combination of Internet-specific rules and reactive restrictions, for instance, the quality and frequency of parent-child communication about social media/internet use.

At a first glance, the finding that less restrictive mediation (i.e Internet-specific rules and reactive restrictions towards Internet use) predicted lower scores on prospective problematic social media use than more restrictive mediation given a supportive parenting context seems to imply that limiting Internet use may even work counterproductive. However, a more plausible interpretation of this finding may be that parents anticipate on their child’s risk for developing problematic social media use. That is, it could be that parents already start limiting their child’s Internet use to a greater extent when they foresee that their child has a higher risk of developing problematic social media use based on other indicators, such as their child’s involvement in other risk behavior or low self-control (prior to actual engagement in problematic social media use; Koning et al., 2013). For future studies it would be interesting to test whether the identified parenting profiles differ in terms of e.g., parental worrying about their child getting addicted to social media or the need to limit the child’s Internet use according to the parent. Besides, to get more insights into whether and how Internet-specific rules and reactive restrictions towards Internet use are effective, future studies should examine whether these parenting practices increase or decrease adolescents’ problematic social media use *within the individual* by e.g., conducting a random intercept cross-lagged panel model.

### The Moderating Role of Adolescents’ Age and Gender

Regarding moderation effects of adolescents’ age and gender, only one significant interaction effect was found using the dataset without participants identified as careless responders; *only younger* adolescents in profile 3 (*Limiting and supportive*) scored lower on problematic social media use at T2 than *younger* adolescents in profile 1 (*Limiting and less supportive*). However, this moderation effect of age is not robust as this effect was only found for one wave. Running the analyses using the dataset including careless responders did not reveal any significant moderation effects. Thus, the relationships between parenting profiles and adolescents’ problematic social media use seem independent of adolescents’ age and gender.

### Limitations

Several limitations of the current study need to be acknowledged. Latent profile analysis is a data-driven approach which means that the profiles identified are affected by sample characteristics. The relatively small sample, which was also quite homogenous in terms of family structure, ethnical background and parental educational level, may limit the generalizability of the present findings. A second limitation is the exclusion of the variable demandingness because of unacceptably low internal consistency. As only two of the three core dimensions of general parenting were included, the current study does not capture all aspects of the general parenting style. A third limitation is that data collection took place during the Covid-19 pandemic in the Netherlands, which may have impacted the results. A large Dutch representative study (HBSC) that showed a significantly higher prevalence of problematic social media use among 12–16-year-olds in autumn 2021 (post-onset pandemic) compared to 2019 and 2017 (pre-pandemic; Boer et al., 2022). Yet, it seems that the pandemic did not influence problematic use in the current study sample as the first measurement wave can be considered as pre-pandemic measure of problematic social media use (as participants were asked to think about the past year and the first measurement wave took place at the very beginning of the Covid-19 outbreak in the Netherlands). Therefore, it is unlikely that the first wave already captured possible influences of the pandemic on problematic use. In addition, the current data showed no significant change in problematic social media use from April/July 2020 to November 2021/January2022. However, parenting practices may have been slightly different than other times as the government-imposed preventive Covid-measures meant a disruption of daily family life. A fourth limitation is that, for several statistical reasons, the classify-analyze approach was used instead of the ML or BCH three-step approach (Nylund-Gibson et al., 2019) when predicting problematic social media use from the latent profiles. A weakness of the classify-analyze approach is that the measurement and estimation error in latent profile membership is ignored which may cause underestimated estimates and standard errors for the effects of latent profile membership on problematic social media use (Nylund-Gibson et al., 2019). However, since entropy (0.75) and average class probabilities (0.85–0.91) were high indicating proper classification of participants in the latent profiles, bias will be limited (Bolck et al., 2004; Clark & Muthén, 2009).

## Conclusion

Existing literature on the relationship between parenting and adolescents’ problematic social media use ignores that parents are likely to apply multiple Internet-specific parenting practices and that these practices happen within and interact with the broader general parenting context. This study examined how different Internet-specific parenting practices (Internet-specific rule setting, reactive restrictions towards Internet use, and co-use) and general parenting dimensions (responsiveness and autonomy-granting) co-occurred and acted together in predicting adolescents’ problematic social media use. The results showed that adolescents who reported relatively high levels of Internet-specific rules, reactive restrictions towards Internet use, responsiveness and autonomy-granting reported less problematic social media use symptoms concurrently and prospectively than adolescents who reported relatively high levels of Internet-specific rules and reactive restrictions towards Internet use, but, at the same time, relatively low levels of responsiveness and autonomy-granting. In addition, adolescents who reported relatively low levels of Internet-specific rules and reactive restrictions towards Internet use, and relatively high levels of responsiveness and autonomy-grating subsequently reported less problematic social media use symptoms than adolescents who reported relatively high levels of Internet-specific rules and reactive restrictions towards Internet use and relatively low levels of responsiveness and autonomy-granting, as well as adolescents who reported relatively high levels of Internet-specific rules, reactive restrictions towards Internet use, responsiveness and autonomy-granting. Thus, it seems that limiting adolescents’ Internet use via rules and reactive restrictions is more effective when parents generally show warmth and acknowledge their children’s opinion. However, not necessarily a certain combination of Internet-specific and general parenting practices, but rather a supportive general parenting context, independent from limiting adolescents’ Internet use via rules and reactive restrictions, seems to play a pivotal role in preventing the risk of problematic social media use. Prevention efforts may benefit most from targeting adolescents living in less supportive parenting contexts as well as informing parents about the importance of a positive parenting climate in preventing problematic social media use among adolescents.
